# Socio-cultural factors favoring home delivery in Afar pastoral community, northeast Ethiopia: A Qualitative Study

**DOI:** 10.1186/s12978-019-0833-3

**Published:** 2019-11-21

**Authors:** Mohammed Ahmed, Meaza Demissie, Alemayehu Worku, Araya Abrha, Yamane Berhane

**Affiliations:** 1 School of Public Health, Samara University, Samara, Ethiopia; 2 Addis Continental, Institute of Public Health, Addis Ababa, Ethiopia; 3 School of Public Health, Addis Ababa University, Addis Ababa, Ethiopia; 4 School of Public Health, Mekelle University, Mekelle, Ethiopia

**Keywords:** Pastoralist, Socio-cultural, Institutional delivery, Home delivery, Ethiopia, Afar

## Abstract

**Background:**

Despite expanding the number of health facilities, Ethiopia has still the highest home delivery services utilization. Health care service utilization varies between regions within the country. This study explored the socio-cultural factors influencing health facility delivery in a pastoralist region of Afar, Ethiopia.

**Methods:**

An explorative qualitative study was conducted in October–December 2015. A total of 18 focus group discussions were conducted separately with mothers, male tribal leaders and religious leaders. In addition, 24 key informant interviews were conducted with Women’s Affairs Bureau and district health office experts and traditional birth attendants and all were selected purposively. Data were coded and categorized using open code software and analyzed based on a thematic approach.

**Results:**

The social factors that affect the choice of delivery place include workload, lack of independence and decision-making power of women, and lack of substitute for childcare and household chores during pregnancy and childbirth. The cultural and spiritual factors include assuming delivery as natural process ought to happen at home, trust in traditional birth attendants, traditional practices during and after delivery and faithful to religion practice, besides, denial by health facilities to benign traditional and spiritual practices such as prayers and traditional food preparations to be performed over there.

**Conclusion:**

Socio-cultural factors are far more than access to health centers as barriers to the utilization of health facilities for child birth. The provision of a maternity waiting home around the health facilities can alleviate some of these socio-cultural barriers.

## Plain English summary

Pregnancy and childbirth-related complications are major causes of deaths and disabilities among the women in sub- Saharan Africa and most of the deaths occur during childbirth. Ethiopia has numerous of health facilities for the maternal health services. However, utilization of the skilled birth attendants in health facilities is very low especially among pastoralists dominated regions of the country. The aim of the study is to explore the socio-cultural factors affecting health facility delivery in a pastoralist region Afar in the northeast, Ethiopia.

A qualitative study comprising eighteen focus group discussions and twenty-four key informant interviews were conducted. Focus group discussions were separately conducted with mothers and male tribal or religious leaders. Key informant interviews were conducted with heads of Women’s Affairs Bureau, district health office heads and traditional birth attendants.

The reasons for preference of majority of women to deliver at home includes home delivery as a natural process, trust in TBA, heavy workload during pregnancy, family support, cultural perceptions, traditional practices and faith in the religion, lack of independence and decision-making power of women and financial dependence on others. The health facilities are also not prepared to cooperate with pastoralist to perform some non-harmful traditional and spiritual practices.

In order to achieve the desired success, strategies should be focused on increasing women awareness about the risks and threats of cultural-traditional practices at home delivery and benefits of the utilization of health facilities.

## Background

A commendable progress has been made over the last several years to improve maternal health worldwide [1, 2]. Even then pregnancy and childbirth-related complications are still potential causes of avoidable deaths and disabilities among women’s at the global level [3, 4]. Almost, all these deaths occur in low-income settings though most of the deaths are preventable [3, 5]. The extent of these maternal deaths due to childbirth-related complications varies among different women population in sub-Saharan Africa, predominantly by low-income population [3, 5].

It indicates the need of developing context-specific strategies to reduce maternal mortality to less than 70 deaths per 100,000 live births by 2030 in all countries and geographic localities [6]. Increasing the institutional delivery with the skilled birth attendant is essential for preventing maternal mortality and has been one of the strategies to achieve the targets set for improving maternal and newborn health [7–9]. Despite the increasing number of health facilities, the utilization of health facilities for delivery is very low in Ethiopia, with considerable variation between the regional states [7, 10].

Ethiopia is a multi-ethnic society, having more than 80 ethnics groups that exercise their own culture and language. A Pastoralist in Ethiopia belongs to more than 29 ethnic groups with an estimated population of 15 million people. The pastoralist population resides in six regional states of Ethiopia that include Somali, Afar, Oromiya, Southern region, Gambella, and Benishangul-Gumuz. These regions account for more than 60% of the total National landmass though they are sparsely populated [11]. The Ethiopian pastoralists community obtains the least benefits from the health sector improvements as much as the agrarian population in the country [12–14].

An Ethiopian demographic health survey recorded 14.7% institutional delivery in Afar compared to Tigray (56.93%), a neighboring predominantly agrarian region [10]. The reasons contributing to the low utilization of institutional delivery were poor health facilities, frequent mobility of pastoralists in search of grazing land and their traditional practices, substantiated by few reports in the literature [13, 15]. Studies conducted in Ethiopia and other developing countries identified the factors as barriers for the utilization of institutional delivery services include inadequate information, insufficient infrastructure, lack of skilled persons and poor quality of services [16–18]. In addition, socio-cultural factors that preclude seeking institutional delivery include trust on Traditional Birth Attendants (TBA) and traditional healers [16, 19, 20], lack of education and knowledge regarding utilization of institutional delivery [20–22], lack of privacy or presence of male birth attendants and inadequate/poor transportation from home to the facility [16, 17, 19, 23, 24].

Since very little is known about the reasons that specifically hinder utilization of institutional delivery in the vast region of Afar, an understanding of the delivery services utilization patterns and the socio-cultural barriers of the Afar pastoralist women can generate useful information to improve institutional delivery services for the pastoralist communities particularly in this country and the continent at large.

## Methods

### Study area

The study was conducted in six districts of Afar regional state of Ethiopia where more than 75% population is living a pastoralist lifestyle. The majority of Afar (Danakil) population is Muslim. Afar region has the lowest altitude in Africa and hot dry climatic conditions that force pastoralist communities to move around constantly in search of grazing land and water. Ethiopia has a three-tier health system consisting of primary health care units which include health centers with satellite health posts at the base, district hospitals in the middle, and specialized hospitals on the top tier. According to Afar regional health bureau report, the region has a total of six hospitals, 86 health centers and 379 health posts. In the study districts, three hospitals, 14 health centers and 69 health posts were functional at the time of the study. All hospitals and health centers provide basic obstetrical services. The health posts do not provide delivery services but refer women in labor to the nearest health center. Each district has an ambulance stationed at the district health office to facilitate referral cases whenever necessary. The ambulance driver has a mobile phone for communication.

### Study design and population

An explorative qualitative study conducted to identify socio-cultural barriers to the utilization of maternal health services. All participants were purposively selected mainly based either on their experience of birth and birth-related traditions or being as important persons. The participants for the focus group discussions (FGD) were the mothers who had children less than 24 months of age, grandmothers and recognized male tribal or religious leaders. A total of 162 individuals participated in 18 FGDs; 60 mothers and 48 grandmothers and 54 male tribal or religious leaders. Key informant interview (KII) participants were consisted of heads of district health office, women’s affairs office and traditional birth attendants who were providing delivery services in the community. Key informant interviews were carried out with a total of 24 participants; six district health office heads, six women’s affairs office heads, and 12 traditional birth attendants (TBAs). TBAs were selected in consultation with health extension workers (HEW), the local health workers, and the women’s association office. The main criteria for their selection were being an active service provider and knowledgeable about the local culture.

### Data collection

Data were collected from October to December 2015. Semi-structured and open-ended FGD and interview guides were developed to guide the data collection. Separate guidelines were prepared and pre-tested conducted with different participants out of study districts in the region. The main focus was on the factors for choosing home delivery. Six midwives a fluent in the local language (Afar) conducted the FGD sessions and key informant interviews after receiving a thorough training. The training provided by the principal investigator was focused on facilitation and interviewing techniques. FGDs and KIIS interviews were conducted in the language and were intimately supervised by the principal investigator. FGDs were conducted in a place agreed by participant and took up to 2 hours while key informant interviews took up to 60 min. The FGDs were conducted by two midwives; one serving as moderator and the other as note taker. Written informed consent was obtained from all participants. All FGDs and KI interviews were recorded using a digital recorder. Audio records were transcribed verbatim and field notes were later integrated into the transcript.

### Data analysis

Audio records were transcribed verbatim and field notes were later integrated into the transcript. The transcribed data were loaded on to the open code software developed by Umea University in Sweden for assisting coding qualitative data [25] and analyzed using Attride-stirling’s framework for thematic network analysis [26]. The transcripts were read repeatedly, coded and organized into categories. Analysis was done using a thematic approach, similar codes were grouped into themes. The result of the thematic analysis is presented in narratives with supporting quotations.

## Results

A total of 186 individuals participated in 18 FGDs; 60 mothers and 48 grandmothers and 54 male tribal or religious leaders. Key informant interviews (KII) were carried out with a total of 24 participants; six district health office heads, six district women’s affairs office heads, and 12 traditional birth attendants (TBAs). All women who participated in the study was housewives with pastoralist lifestyle. All participants were ever married and Muslims and mostly (89%) were unable to read and write. Most of the women, 47 out of the 60 had their recent delivery at home assisted by traditional birth attendants. The remaining gave birth at health facility either due to birth complications (9 women) or preferred to deliver at a health facility (4 women). Among the 24 key informants from district offices, 6 were the males and 18 females. The TBAs reported at least 6 years of experience in providing delivery services in the area, however, all were unable to read and write and Muslim by faith. (Table 1).

**Table 1:** Socio-demographic characteristics of the focus group dissuasion (FGD) and Key informant participants (KII)

Socio-demographic traits	FGD Participant		KII Participant		
	Mothers (60) and grandmothers (#48)	Male tribal or religious leaders (#54)	District health office heads, (#6)	women’s affairs office heads(#6)	Traditional birth attendants(#12)
Age					
Below 30 years	52 (48%)	2 (4%)	1 (17%)	0	0
30 or above	56 (52%)	52 (96%)	5 (83%)	6	12
Religion					
Islam	108 (100%)	54 (100)	6	6	12
Christina	0	0	0	0	0
Education level					
Illiterate	96 (89%)	39 (72%)	0	0	12
Primer education	11 (10%)	12 (22%)	0	0	0
Secondary and more	1 (1%)	3 (6%)	6	6	0
Occupation					
Home duties and pastoralist	108 (100%)	0	0	0	0
Pastoralist	0	42 (78%)	0	0	0
Gov employee	0	0	6	6	0
Self-employed (Business)	0	12 (22%)			12 (TBA)
Daily labor	0	0	0	0	0
Marital status					
Married	89?(82.4)	50 (93%)	6	6	9
Widowed	15 (13.9%)	4 (7%)			3
Divorced	4 (3.7%)	0	0	0	0
Birth place (last pregnancy for 60 women)					
Home	92 (85.1%)	0	5	1 (17%)	11 (92)
Health facility	16 (14.8%)	0	0	5 (83%)	1 (8)

### The social factors for home delivery preference

#### Women’s heavy workload

In the pastoralist community, women are engaged in household chores throughout the day to meet their basic daily needs and of children and husband. In comparison to male members of the family, pastoralist women avail much less resting time. Same is true during pregnancy, working almost until their delivery time. Women work from dawn to dusk, their daily chores include fetching and carrying water and firewood from distant place, grazing animals, child care and food preparation. They have no time to relax even during pregnancy. The following quote illustrates the situation

*“In our village, we normally do not go to a health facility for delivery because we cannot be away from the family due to the excess workload we shoulder daily and the family is entirely dependent on us for household matters … we do not want the family to be in trouble … so, we work until delivery”. (FGD woman n participant, 45 years old)*

A pastoralist woman, in some instances, would be compelled to give birth while working in the field. That happens because most women do not know the expected delivery date, therefore continues strenuous work until they actually deliver rather unexpectedly.

*A participant said, “many women start labor pain whilst away from home to conduct their routine work often ending with an unexpected delivery in the field. You know, some women also experience a very short or no labor pain preceding the delivery of a baby, which makes them deliver quickly in the field”. (FGD woman participant,23 years old)*

*… Women often go to a health facility for a delivery with complications such as a ruptured membrane (no water), bleeding or insufficient or no power to push the baby out”. (KIIs TBA participant, 65 years old).*

#### Perceiving delivery as a normal process and trust on TBAs

Majority of the woman participants considers delivery as a normal process which can be conducted at home safely under the supervision of TBA. TBA’s are acceptable to the community because of their easy access, sharing of common cultural values and their ability to converse in the local language. Women consider TBAs more approachable and helpful. Due to these reasons pastoralist women prefer home as the first choice for a child birth. The following is a quote from a young mother:

*“We believe the TBAs have enough experience and ability to handle all delivery cases smoothly … they are familiar with the types of complication related to delivery and the solutions too. When labor starts, women usually call the TBA who assisted and managed welled in previous deliveries in the neighborhood”.*

*(FGD Woman participant, 29 years old)*

*“TBAs help and instruct us likes mothers … they have techniques to hasten the birth process and suggest a suitable position. For women who have no relative at home, they cook food and help the family by doing home chores … they collect firewood, manage domestic animals, and wash the women’s clothes after delivery”. (FGD Woman participant, 22 years old)).*

Community and religious leaders have the same opinion on this issue like women. They think pregnancy and delivery are natural processes that do not require any special medical attention,and peer mothers or TBAs can provide the required care at home. They presume, women referred to health facilities would be subjected to unnecessary surgical intervention to deliver a baby.

*“As a woman giving birth at home is good, naturally they should be able to carry the pregnancy and have a normal delivery assisted by a TBA, that is what is expected. women referred to the hospital mostly have surgery (cesarean section)”. (KIS Male participant, 59 years old).*

Women have also expressed their opinion regarding preference to seek help from a TBA for home delivery due to the desire to have customary traditional practices that need to be performed by TBAs such as female circumcision and suturing genitalia. TBAs refuse to do these harmful but still desirable practices for babies whose mothers gave birth, not under their supervision.

Women said …” … *failure to have traditional practices such as female circumcision and genital suturing causes a serious problem for female babies. If delivery is conducted at a health facility, TBAs are not willing to do the suturing afterward and without having done circumcision and suturing, women will not be considered as a suitable wife … a cause of difficulty to find a husband for them in the future”.(FGD Woman participants,52 years old)*

#### Family support

Women mentioned that the presence of family members, such as mothers, husband or grandmother, before and after the delivery process provides a psychological support and encouragement in the laboring woman. Family members also care for their children and create a conducive environment at home during delivery. Such support is not available at health facilities. Family members cannot visit laboring mothers in the facility due to restrict access to health facilities lack of transport, and a place to stay around the facility besides meager availability of food around health centers. Most women prefer to deliver at home

*“I like delivering at home because my mother will stay with me during the labor and I also feel more secure and fearless when the family is with me”. (FGD woman participant, 29 years)*

*“I feel more comfortable with home delivery … I see my neighbors and friends taking care of my children and animals … that makes me relaxed and the process less stressful”. (KIS woman head participant, 29 years)*

### Cultural perception, traditional beliefs and practices and faith in religion

In a pastoralist community, people are firmly attached to certain cultural and traditional practices and rituals. They feel proud to execute these practices as they are an inseparable part of their pastoralist lifestyle. The choice about where a woman would give birth is made by family members and relatives. They usually get together to pray at home for the laboring mother. Key informants believe that spiritual and cultural practices are among the reasons of not delivering at health facilities. There are also certain practices which are supposed to be done at home by elders immediately after the birth. Such practices can be performed only at home and it may as allowed in the health facilities.

. “*It is our tradition to give the newborn baby “onqor’ (drops of a mixture of milk, water, honey or herbal medicines) immediately after birth. In addition, ‘Azan’ (Islamic prayer) is practiced immediately after birth. Both these practices are not allowed in health facilities”. ((FGD Woman participant, 25 years old)*

“*… no matter how poor or rich you are, you have to receive the newborn with warm ululation upon delivery … we also prepare special traditional food like ‘Asido’ and ‘Alko-Hado’ immediately after delivery and eating together that special food is something which no one wants to miss”. Other cultural practices after delivery include hot baths, hot Surba (soup) and heated bedding … we keep the fire going in the house to make the sleeping place (‘Boodo’) warm … that helps to remove dirty blood from the mother’s system”.(A 53-year-old male in one of the FGD)*

The women also complained about the improper disposal of the placenta at health facilities in toilet hole or other places, which are traditionally unacceptable. Placenta must be buried properly in the backyard at home. People also believe the umbilical cord must be cut and a stitched using a traditional knife (*Makita)*, and suturing material.

*Women said “In health facilities, they (health care providers) throw the placenta just anywhere … that is not the right way of disposing a placenta … in our culture, we cover it with a piece of cloth, and bury it in a well-protected pit … proper handling of the placenta is important … we believe that will keep the child thriving in his village/community”. (FGD Woman participants 25 years old)*

Traditional women also feel shy or a shamed in discussing their obstetric problems or symptoms with a male health care provider. The study participants mentioned that women, in general, are uncomfortable to have maternity care from male health care providers.

*“We do not like to expose our naked body for ‘Agnabes” (male who is not the husband); it is only my husband who has the right to see me bare. It is “harem” (forbidden by God), Allah will not forgive me and, in truth, it is disagreeable (culturally) and intolerable in Afar to expose my private part to a male provider, therefore we prefer to deliver at home”. (FGD participant, 29 years old mother)*

### Lack of women decision-making power

Women in Afar generally have very little decision-making power even regarding where and when to seek-health care during pregnancy and childbirth. In the community under study, such decision-making power is vested in a network of close linked people that consist of the husband, his clan members, mother/mother-in-law, and TBAs. They have the ultimate authority to take a final decision about the pregnant mother’s health seeking practice and/or choose a place of delivery/childbirth.

The woman participants said, “*… when my time to give birth approached, my husband decided that I should give birth at home. My mother-in-law also supported his decision and persuaded me to give birth at home by telling her story that she gave birth to all her children at home and never had any problem … she did not want me to go to a health center”. (FGD Woman participant, 25 years old)*

*Another woman said,* “*… husbands’ make a decision on the place of delivery, if he (the husband) is not at home, his clan members or his mother-in-law decide whether the woman should go to a health facility or not”.(FGD participant, 35 years old woman)*

The majority of participants in FGD and KIs indicated women in the region generally lack the power of independent decision-making to seek health care and have no equal rights to the family resources.

In this community, even though women generate sufficient income contributing to a greater portion of household economy has no rights spend it without the consent of their husband.

*“We are living in the community where the culture of male dominance and female submissiveness is deeply rooted … women dependence on men has created an unequal power relationship between men and women, and that usually deny women access to beneficial social services. “.(KIs head of women affair office)*

Participants of both FGDs and KIs also indicated that pastoralist women may also suffer from freedom of movement outside their villages; Afar women often stay in the villages and handle household affairs. Even if she has sufficient money, she has no information on the available health care options and has to rely on her husband’s decision.

Another woman said that,

*“Women always stay at home caring for children, we are not allowed to move outside the village and thus we know very little about facilities available outside our village … how we can make a decision to give birth in a health facility … that is why we deliver at home”. (FGD Woman participants 35 years old)*

## Discussion

This study revealed the importance of various socio-cultural barriers on the use of health facility for childbirth by the women of pastoralist community predominantly living in Afar region of Ethiopia. Some factors such as the lack of place to perform prayers and cultural rituals at facilities, lack of confidence in delivery care providers, avoiding male delivery attendants in health facilities, and improper disposal of the placenta can be overcome By considering the of guidelines and training for service providers. Some factors such as lack of autonomy in women and their desire to continue traditional practices which are deeply-rooted require strong community engagement. For instance, the desire to carry on traditional and cultural rituals such as special meals after delivery, having prayers during labor, and traditional way of placenta burial are such practices and rituals that favor home delivery. In addition, women’s desire to have traditional disposal of placenta and issues related to female genital mutilation practices are also other important barriers to decide the choice for institutional delivery. These findings are in agreement with other studies conducted in Ethiopia [27, 28]. These practices are usually recognized as an identity belonging to a particular socio-cultural group and women are unwilling to give it up as also shown by other studies [15]. The health system needs to introduce some cultural practices in their future strategies to improve utilization of services [22].

The most frequent reason for preferring home delivery is a feeling of shame to deliver in presence of a male attendant in the health facility and lack of privacy at the health facility makes women uncomfortable by exposing their private parts during delivery. These are among others important reasons for the under utilization of delivery services recorded in similar studies conducted in Ethiopia and other countries as well [15, 18, 23, 29]. Training and assigning culturally competent female midwives/delivery assistants is necessary for such settings where women are forbidden to show their private body to non-partner males.

Our study also elucidates those practices which are supposed as done at home by elders immediately after the birth important reasons to home delivery. This can be achieved by providing maternity waiting areas that allow bringing of family members closer to the delivery place. Other studies also report offering maternity waiting area that can help to create an environment that simulates home feelings and help overcome the physical and cultural barriers that discourage health facility delivery [30, 31]. Although maternity waiting areas near facility centers have been introduced in Ethiopia several years ago [32] and seen as effective in reducing maternal death and stillbirth rates [31, 33], they have not been widely established in pastoralist inhabitations.

Our findings showed that women lack confidence in birth attendants at facilities. Women perceive that health providers lack cultural competence and skills to properly assist the delivery. However, these findings are not unique to this study area, and there are common reasons why women prefer home delivery unless the labor has complications [17, 18]. Similar findings have been reported from other countries [34].

According to our study results, great majority participants perceived home delivery as a normal process easily assisted by traditional birth attendants and believe TBAs have enough longtime experiences to handle all delivery and also, they expressed that because of access, familiarity, cultural values and help mothers cook food, household work and manage domestic animals. This is in line with previous study’s birth preferences were due to TBA have experiences and services obtained without spending of cost [15, 23].

As most of the study participants mentioned that, pastoralist women do not go to a health facility for delivery because they remain engaged in household chores throughout the day to meet their basic daily needs and of children and husband during pregnancy, working almost until their delivery time. Studies conducted in other countries have also recognized that heavy work; load and childcare are responsible for under utilization of .health facilities [20, 35, 36]. Another study in Gambia shows that as a result of their excessive workload, was a reason for their inability to access health facility care at the onset of labor and their preference for birthing at home under the supervision of a traditional birth attendant [37].

Another important reason for giving birth with a TBA has proved that the women in our study area assume that they will be subjected to unnecessary surgical intervention to deliver a baby. This concern also contributes to women’s fear of going to a facility and their preference for home delivery at home. This finding is supported by study conducted in African, which founds those women who had undergone a caesarian section were afraid of having another and as a result, intended for their next delivery to be at home [38].

Our study also elucidates the importance of decision-making process about where to give birth, women in the community generally lack the power of independent decision making to seek health care and have no equal rights to the family resources. Research conducted in Sub-Saharan Africa’s countries also recognized lack of women’s autonomy to seek care, and economic dependency on their husbands as crucial barriers for institutional delivery [20, 39]. In pastoralist communities, husbands often do not stay at home, thus the decision to seek care is made collectively by the mother-in-law and clan members in consultation with the TBAs who often favor health institution delivery only when they perceive life-threatening complications [16, 17, 40].

The study amply describes the context and important barriers hampering institutional delivery in pastoralist community including the social factors, cultural perception and traditional practices in rural pastoralist areas. Measures to provide desired health information and community awareness on pregnancy and childbirth care should be the focus of the policymakers to develop a knowledgeable society for taking timely maternal health-related decisions, possibly among other factors through capacity building, increase in woman midwifery services, proper training of TBA’S about the risks of pregnancy, hygiene & cleaning condition and adequate referral system. This may attract pregnant women to prefer institutional delivery system.

The strengths of this study carried out in a challenging research environment, and among the few studies conducted in pastoralist communities in Ethiopia. Including male partners/husbands and health workers in the interview might have given a different opinion on the issue, and is one of the limitations of this study. A quantitative study with a representative, adequate, and sample size based on the findings of this study would give more comprehensive information to plan interventions that will enhance the utility of skilled birth attendants.

This study was focused on community, to determine reasons of under utilization of delivery services. Further study is needed to conduct more researches to understand health workers serving in public health care facilities perception for under utilization and to find mechanism for the improvement of women and child health.

## Conclusions

This study indicated that socio-cultural factors are strong barriers to utilization of institutional delivery services in the Afar Pastoralist community. Overcoming deep-rooted socio-cultural practices require sustained community dialogues and considering harm reducing strategies to improve the utilization of delivery services.

## Data Availability

The data used in the current study was made available from the corresponding authors on reasonable request.

## Abbreviations

FGD: Focus group discussions
KII: Key informant interview
HEW: Health extension workers;
TBAs: TBAs traditional birth attendants
